# Association between possession of ExoU and antibiotic resistance in *Pseudomonas aeruginosa*

**DOI:** 10.1371/journal.pone.0204936

**Published:** 2018-09-28

**Authors:** Dinesh Subedi, Ajay Kumar Vijay, Gurjeet Singh Kohli, Scott A. Rice, Mark Willcox

**Affiliations:** 1 School of Optometry and Vision Science, University of New South Wales, Sydney, Australia; 2 The Singapore Centre for Environmental Life Sciences Engineering, Nanyang Technological University, Singapore; 3 The School of Biological Sciences, Nanyang Technological University, Singapore; 4 The ithree institute, The University of Technology Sydney, Sydney NSW Australia; Laurentian, CANADA

## Abstract

Virulent strains of *Pseudomonas aeruginosa* are often associated with an acquired cytotoxic protein, exoenzyme U (ExoU) that rapidly destroys the cell membranes of host cells by its phospholipase activity. Strains possessing the *exoU* gene are predominant in eye infections and are more resistant to antibiotics. Thus, it is essential to understand treatment options for these strains. Here, we have investigated the resistance profiles and genes associated with resistance for fluoroquinolone and beta-lactams. A total of 22 strains of *P*. *aeruginosa* from anterior eye infections, microbial keratitis (MK), and the lungs of cystic fibrosis (CF) patients were used. Based on whole genome sequencing, the prevalence of the *exoU* gene was 61.5% in MK isolates whereas none of the CF isolates possessed this gene. Overall, higher antibiotic resistance was observed in the isolates possessing *exoU*. Of the *exoU* strains, all except one were resistant to fluoroquinolones, 100% were resistant to beta-lactams. 75% had mutations in quinolone resistance determining regions (T81I *gyrA* and/or S87L *parC*) which correlated with fluoroquinolone resistance. In addition, *exoU* strains had mutations at K76Q, A110T, and V126E in *ampC*, Q155I and V356I in *ampR* and E114A, G283E, and M288R in *mexR* genes that are associated with higher beta-lactamase and efflux pump activities. In contrast, such mutations were not observed in the strains lacking *exoU*. The expression of the *ampC* gene increased by up to nine-fold in all eight *exoU* strains and the *ampR* was upregulated in seven *exoU* strains compared to PAO1. The expression of *mexR* gene was 1.4 to 3.6 fold lower in 75% of *exoU* strains. This study highlights the association between virulence traits and antibiotic resistance in pathogenic *P*. *aeruginosa*.

## Introduction

*Pseudomonas aeruginosa* infections can be severe in people with a compromised immune system and impaired anatomical structures caused by, for example burns, cystic fibrosis or mechanical abrasions [1]. *P*. *aeruginosa* is a successful opportunistic pathogen in part due to its production of a diverse repertoire of pathogenic factors and its innate ability to evade the host immune system [2]. Treatment of *P*. *aeruginosa* infections can be challenging due to the inherent antibiotic resistance, where some studies have shown that half of the isolates from clinical infections were resistant to antibiotics [3]. Furthermore, reports on co-selection of antibiotic resistance and pathogenic factors indicate that antibiotic resistance may be a factor for the evolution of more virulent strains of *P*. *aeruginosa* or *vice versa* [4–13].

Many Gram-negative bacteria, including *P*. *aeruginosa*, possess type III secretion systems (TTSS), which they utilise to introduce virulence factors directly into host cells [14]. In *P*. *aeruginosa*, TTSS transports four secreted factors: ExoU, ExoS, ExoY and ExoT. However, all of these factors may not be common in all *P*. *aeruginosa* strains. For example, the *exoS* gene was present in 58–72%, the *exoU* gene in 28–42%, the *exoY* gene in 89% and the *exoT* gene in 92–100% of isolates from acute infections [15]. Pathogenic strains contain either *exoU* or *exoS*, but rarely both [16, 17]. The *exoU* gene is associated with a genomic island and its acquisition may cause loss of the *exoS* [18, 19]. The *exoU* gene encodes a cytotoxic protein that rapidly destroys the cell membranes of mammalian cells by its phospholipase activity [19]. The presence of *exoU* correlates with phenotypes that are responsible for the severe outcome of many infections including pneumonia [20] and keratitis [21]. Up to two-thirds of ocular isolates of *P*. *aeruginosa* possess the *exoU* gene [22], which is a much higher rate than the isolates from other infections [6, 23, 24].

The frequency of antibiotic resistance of the *exoU* gene carrying strains is higher than that of *exoS-*strains; [5, 10] the reason for this higher frequency remains undefined. *P*. *aeruginosa* strains with the *exoU* gene tend to harbour mutations in quinolone resistance determining regions (QRDRs) that lead to fluoroquinolone resistance [5, 9]. Whilst it is known that strains of *P*. *aeruginosa* can possess mutations in resistance determining regions affecting beta-latam susceptibility, such as the chromosomal beta-lactamase gene (*ampC*), its transcriptional regulator (*ampR*) [25] and a repressor gene (*mexR*) that negatively regulates expression of an active efflux pump (MexAB-OprM) [26], the correlation between the *exoU* carriage and mutations in drug resistance determining regions has not been extensively examined.

We hypothesised that possession of the *exoU* gene correlates with mutations not only in QRDRs but also in beta-lactam resistance determining regions. The aim of this study was to examine the correlation between the virulent genotypes (*exoS* vs. *exoU*) and resistance to beta-lactam and fluoroquinolone antibiotics in *P*. *aeruginosa* strains. Furthermore, we examined the relative expression of specific genes to confirm their role in antibiotic resistance.

## Materials and methods

### Bacterial isolates and antibiotic susceptibility testing

Twenty-two *P*. *aeruginosa* strains isolated from anterior eye infections, microbial keratitis (MK), or lungs of cystic fibrosis patients from India and Australia were used in this study (Table 1). The minimum inhibitory concentrations (MICs) of ceftazidime (Sigma-Aldrich, Inc., St. Louis, MO, USA), cefepime (European Pharmacopoeia, Strasbourg, France) aztreonam (Sigma-Aldrich, Inc), ticarcillin (Sigma-Aldrich, Inc), imipenem (Sigma-Aldrich, Inc), levofloxacin (Sigma-Aldrich, Inc), ciprofloxacin (Sigma-Aldrich, Inc), and moxifloxacin (European Pharmacopoeia) were determined by the broth microdilution method as described by the Clinical and Laboratory Standards Institute (CLSI) [27]. The MIC was taken as the lowest concentration of an antibiotic in which no noticeable growth (turbidity) was observed [28] and the break point was established according to published standards [29, 30]. Both resistant and intermediate resistant strains were considered here as resistant.

**Table 1:** Strains and origin of *Pseudomonas aeruginosa* used in this study.

Isolate designation	Origin	Associated infections
PA17	Australia	MK
PA149	Australia	MK
PA157	Australia	MK
PA171	Australia	MK
PA175	Australia	MK
PA40	Australia	MK
PA32	India	MK
PA33	India	MK
PA34	India	MK
PA35	India	MK
PA37	India	MK
PA82	India	MK
PA55	Australia	CF
PA57	Australia	CF
PA59	Australia	CF
PA64	Australia	CF
PA66	Australia	CF
PA86	Australia	CF
PA92	Australia	CF
PA100	Australia	CF
PA102	Australia	CF
PAO1	Reference strain [64] (RefSeq accession no. NC_002516.2)	

MK = Microbial keratitis, CF = Cystic fibrosis

### DNA extraction and sequencing

Bacterial DNA was extracted from overnight cultures grown on Trypticase Soy Agar (TSA; Oxoid Ltd., Basingstoke, UK), using the DNeasy Blood and Tissue Kit (Qiagen, Hilden, Germany) following the manufacturer’s instructions. The extracted DNA was sequenced on MiSeq (Illumina, San Diego, CA, USA) platform generating 300 bp paired-end reads. The paired-end library was prepared using Nextera XT DNA library preparation kit (Illumina, San Diego, CA, USA). All of the libraries were multiplexed on one MiSeq run. Genome assembly and annotations were performed using SPAdes version 3.11.1 [31] and Prokka version 1.7 [32]. BLAST search was performed to investigate carriage of *exoU* and *exoS* genes. All nucleotide sequences were deposited in NCBI GenBank data base under Bio-project accession number PRJNA431326.

### Sequence analysis and variant calling

The mutations in selected resistance genes (*gyrA*, *gryB*, *parC*, *parE*, *mexR*, *ampC*, *ampD* and *ampR*) of each strain were determined with reference to *P*. *aeruginosa* PAO1 (Genbank RefSeq accession no. NC_002516.2). Briefly, the reference genome was mapped to the paired-end reads for each isolate using Bowtie2 version 2.3.2 [33] and the variants were compiled and annotated using SAMtools, version 1.7 [34] and SnpEff version 4.3 [35]. The QRDRs were assigned to amino acid positions 83 to 87 of the GyrA protein, positions 429 to 585 of the GyrB protein, positions 82 to 84 of the ParC protein, and positions 357 to 503 of the ParE protein [36]. For *ampC* variants, mutations different from common polymorphisms (G27D, R79Q, T105A, Q156R, L176R, V205L, and G391A), which are present in both susceptible and non-susceptible strains [37] were considered here. Mutations in *mexR* or *ampR* were considered as significant for resistance from previous literature [38–40].

### Total RNA extraction and qRT-PCR analysis

Strains were revived from frozen stocks into 5 mL Trypticase Soy Broth (TSB; Oxoid) and grown to mid-exponential phase (OD_660_ 1.5) and 1 ml was centrifuged at 6000 *g* for 3 min to harvest the cells. The pellet was mixed with 1 mg/ml lysozyme in Tris-EDTA buffer (TE; 10 mM Tris-hydrochloride and 1.0 mM EDTA pH 8.0) to lyse the cells. RNA extraction was performed using the ISOLATE RNA Mini Kit (Bioline, London, UK) following the manufacturer instructions for RNA isolation from bacteria. The RNA extract was treated with DNase1 (Bioline) to eliminate the DNA contamination and purified by ethanol precipitation [41]. RNA purity and concentration was measured by NanoDrop spectrophotometer (ND-1000, ThermoFisher, MA, USA). cDNA was synthesized from 1 μg of total RNA using SuperScript First-Strand synthesis system for RT-PCR (Invitrogen, Carlsbad, CA, USA) employing random primers and following the manufacturer’s protocol. Quantitative PCR was performed with a PowerUp SYBR Green Master Mix (Applied Biosystems, Austin, TX, USA), using 96 well optical plates (MicroAmp Fast Optical, Applied Biosystems) following the manufacturer’s instructions and cycle conditions. A 7500 Fast Real-Time PCR System (Applied Biosystems) was used to measure the expression levels of the target DNA sequences using gene specific primers (Table 2). The relative expression levels were quantified using the comparative C_T_ method [42] to obtain the fold change in each gene with reference to the respective genes of *P*. *aeruginosa* PAO1, which does not carry the *exoU* gene. A house keeping gene, *rpsL* encoding the 30S ribosomal protein S12, was used as an internal expression control for normalisation. The experiments were carried out three times in triplicate and the mean and standard deviations were calculated.

**Table 2:** Primers used in this study.

Genes	Functions	Primers (5–3)	Length (bp)	Nucleotide position in gene	Product length (bp)	References
*ampC*	Cephalosporinase	CGGCTCGGTGAGCAAGACCTTC–F	22	264	218	[46]
		AGTCGCGGATCTGTGCCTGGTC–R	22	481		
*mexR*	Transcriptional regulator	CGCGAGCTGGAGGGAAGAAACC–F	22	217	150	[46]
		CGGGGCAAACAACTCGTCATGC–R	22	366		
*ampR*	Transcriptional regulator	TGCTGTGTGACTCCTTCGAC–F	20	215	160	This Study
		AGATCGATGAAGGGATGGCG–R	20	374		
*rpsL*	30S ribosomal protein S12 (house keeping gene)	GCAAGCGCATGGTCGACAAGA–F	21	35	201	[46]
		CGCTGTGCTCTTGCAGGTTGTGA–R	23	235		

## Results

### Possession of *exoU/exoS* and antibiotic resistance

**Fig 1:**
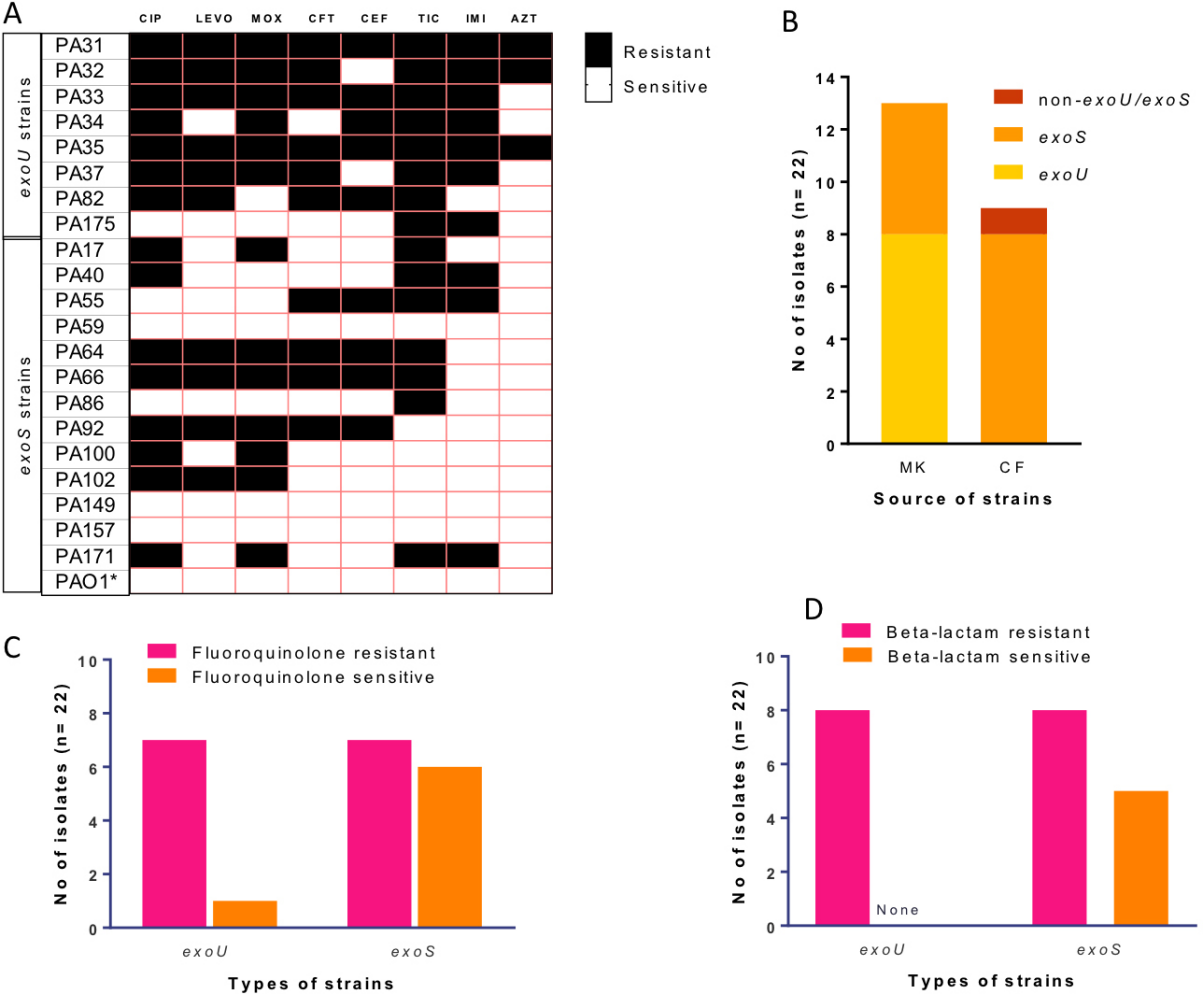
Antibiotic susceptibility patterns and possesion of *exoU* and *exoS* genes. **A)** Antibiotic susceptibility pattern of *exoU* and *exoS* strains. Both resistant and intermediate resistant strains were considered here as resistant. Black boxes represent resistance and white boxes represent susceptibility. **B)** Number of microbial keratitis (MK) and cystic fibrosis (CF) isolates that carry *exoU* or *exoS* genes. **C)** susceptibility of *exoU* and *exoS* strains to fluoroquinolones. **D)** susceptibility of *exoU* and *exoS* strains and to beta-lactams. [CIP = ciprofloxacin, LEVO = Levofloxacin, MOX = Moxifloxacin, CFT = ceftazidime, CEF = cefepime, TIC = ticarcillin, IMI = imipenem, AZT = Aztreonam].

BLAST search showed that of the 22 isolates, 8 out of the 13 eye isolates (62%) possessed the *exoU* gene, while it was absent from the cystic fibrosis (CF) isolates (Fig 1B). Except for a CF strain (PA57) which lacked both *exoU* and *exoS* genes, all strains that lacked the *exoU* gene carried the *exoS* gene and none of the studied strains harboured both genes. For the *exoS* strains, 8/13 showed a medium level (2–16 μg/ml) of resistance to at least one fluoroquinolone and 8/13 were resistant to at least one beta-lactam, mostly ticarcillin (Fig 1A). Six of these eight strains were resistant to both a fluoroquinolone and beta-lactam. All except one (PA175) *exoU* strain were resistant to at least two tested fluoroquinolones with MICs of between 2–128 μg/ml with six strains having ≥32 for ciprofloxacin. All the *exoU* strains were resistant to at least three beta-lactams except for PA175, which was only resistant to ticarcillin and imipenem (Fig 1B, 1C and 1D).

### Mutations in target genes of QRDRs and possession of *exoU*

Mutations in four different QRDRs were examined with reference to *P*. *aeruginosa* PAO1. Of the strains containing *exoU*, 6/8 had a T83I mutation in *gyrA* and 5/8 had combined mutations in both *gyrA* (T83I) and *parC* (S87L); none of the *exoS* strains had either of these mutations. Strains with mutations in *gyrA* or *parC* were resistant to all three fluoroquinolones and these mutations correlated with higher MICs for fluoroquinolones (Table 3). None of the *exoS* strains possessed mutations in *gyrA* and *parC*. However, mutations were observed in *gyrB* and *parE* in five *exoS-*strains which were associated with higher MIC to fluoroquinolones (Table 3). Interestingly, no mutations in *gyrB* or *parE* were found in the *exoU* strains. It should be noted that five strains (PA82, PA17, PA40, PA100 and PA171) had no mutations in any of these genes, but were resistant to at least one fluoroquinolone, although resistance tended to be ≤8 μg/ml, except PA82 which had an MIC of 64 μg/ml for ciprofloxacin.

**Table 3:** Mutations in the quinolone resistance determining region of *P*. *aeruginosa* and the MIC of fluoroquinolones.

	Strain	Genes				MIC (μg/ml) of Fluoroquinolones		
		*gyrA*	*gryB*	*parC*	*parE*	CIP	LEVO	MOX
*exoU* strain*s*	PA31	T83I		S87L		**32**	**32**	**64**
	PA32	T83I		S87L		**64**	**32**	**128**
	PA33	T83I		S87L		**128**	**32**	**64**
	PA34					**2**	2	**8**
	PA35	T83I		S87L		**64**	**32**	**128**
	PA37	T83I		S87L		**64**	**32**	**128**
	PA82					**64**	**4**	4
	PA175	T83I				0.25	0.25	1
*exoS* strain*s*	PA17					**2**	1	**8**
	PA40					**4**	2	2
	PA55					1	0.5	4
	PA59					0.125	0.25	1
	PA64				A473V	**8**	**16**	**16**
	PA66		E468D			**2**	**4**	**8**
	PA86		L457-458A*			0.5	1	2
	PA92		S466F		A473V	**4**	**4**	**16**
	PA100					**2**	1	**8**
	PA102				A473V	**2**	**4**	**8**
	PA149					0.5	0.5	2
	PA157					0.25	0.5	2
	PA171					**4**	2	**8**
	PAO1**					0.25	0.25	1

The numbers denote change in amino acid positions when compared with the genome of *P*. *aeruginosa* PAO1.

**Bold** = resistant or intermediate resistant.

CIP = ciprofloxacin, LEVO = Levofloxacin, MOX = Moxifloxacin.

*Insertion and frameshift variant.

** Reference strain

### Mutations associated with beta-lactam antibiotics and *exoU*

This study also examined mutations in cephalosporinase (*ampC*) and its regulator (*ampR*) and the efflux pump MexAB-OprM regulator (*mexR*). A number of mutations were seen in *ampC*, but only those mutations that have been previously reported to be significant contributors to resistance were considered here (all observed mutations are shown in S1 Table). Variation in amino acid position V356I was common in six *exoU* strains, Q155R was found in strain PA82 and no significant mutations were observed in PA34. Such mutations were, however absent in *exoS-*strains. Similarly, all of the *exoU* strains had a common mutation in the in *mexR* gene at amino acid position 126, changing valine to glutamic acid, in addition to mutations at A110T in PA34 and K76Q in PA175. Such mutations were not present in the *exoS* strains. Furthermore, all the *exoU* strains had various mutations (E114A, G283E, and M288R) in the *ampR* gene, but only one *exoS* strain (PA171) had mutations in this gene and this at position 244. The susceptibility results showed that possession of such mutations was associated with higher MICs to various beta-lactams, except for strain PA175 which had all these mutations in *mexR*, *ampC*, and *ampR* genes but was sensitive to cefepime and ceftazidime (Table 4).

**Table 4:** Mutations in beta-lactam resistance determining regions and beta lactam resistance profiles.

	Strains	Genes			MIC (μg/ml) towards beta-lactams					
		*mexR*	*ampC*	*ampR*	CFT	CEF	TIC	IMI		AZT
*exoU* strains	PA31	V126E	V356I	G283E, M288R	**16**	**16**	**64**	**4**		**16**
	PA32	V126E	V356I	G283E, M288R	**16**	8	**64**	**4**		**32**
	PA33	V126E	V356I	G283E, M288R	**32**	**16**	**128**	**8**	8	
	PA34	A110T, V126E		E114A, G283E, M288R	4	**32**	**>128**	**16**	8	
	PA35	V126E	V356I	G283E, M288R	**16**	**32**	**128**	**8**	**16**	
	PA37	V126E	V356I	G283E, M288R	**16**	8	**64**	**4**	8	
	PA82	V126E	Q155I	G283E	**>128**	**>128**	**32**	1	8	
	PA175	K76Q, V126E	V356I	G283E, M288R	2	4	**64**	**4**	8	
*exoS* strains	PA17				4	4	**128**	1	8	
	PA40				1	2	**64**	**4**	8	
	PA55				**32**	**64**	**32**	**4**		8
	PA59				2	2	16	1		8
	PA64				**16**	**64**	**32**	1		4
	PA66				**16**	**64**	**32**	0.25		8
	PA86				4	8	**64**	1		8
	PA92				**32**	**64**	8	1		1
	PA100				4	8	16	2		0.5
	PA102				1	1	16	0.5		4
	PA149				2	4	16	1		4
	PA157				2	4	16	1		4
	PA171			R244W	2	1	**32**	**4**		8
	PAO1**				1	1	16	1		4

The numbers denote change in amino acid positions when compared with the genome of *P*. *aeruginosa* PAO1.

Bold = resistance or intermediate resistance.

CFT = ceftazidime, CEF = cefepime, TIC = ticarcillin, IMI = imipenem, AZT = Aztreonam.

**Reference strain

### Expression analysis of *ampC*, *ampR* and *mexR* genes

**Fig 2:**
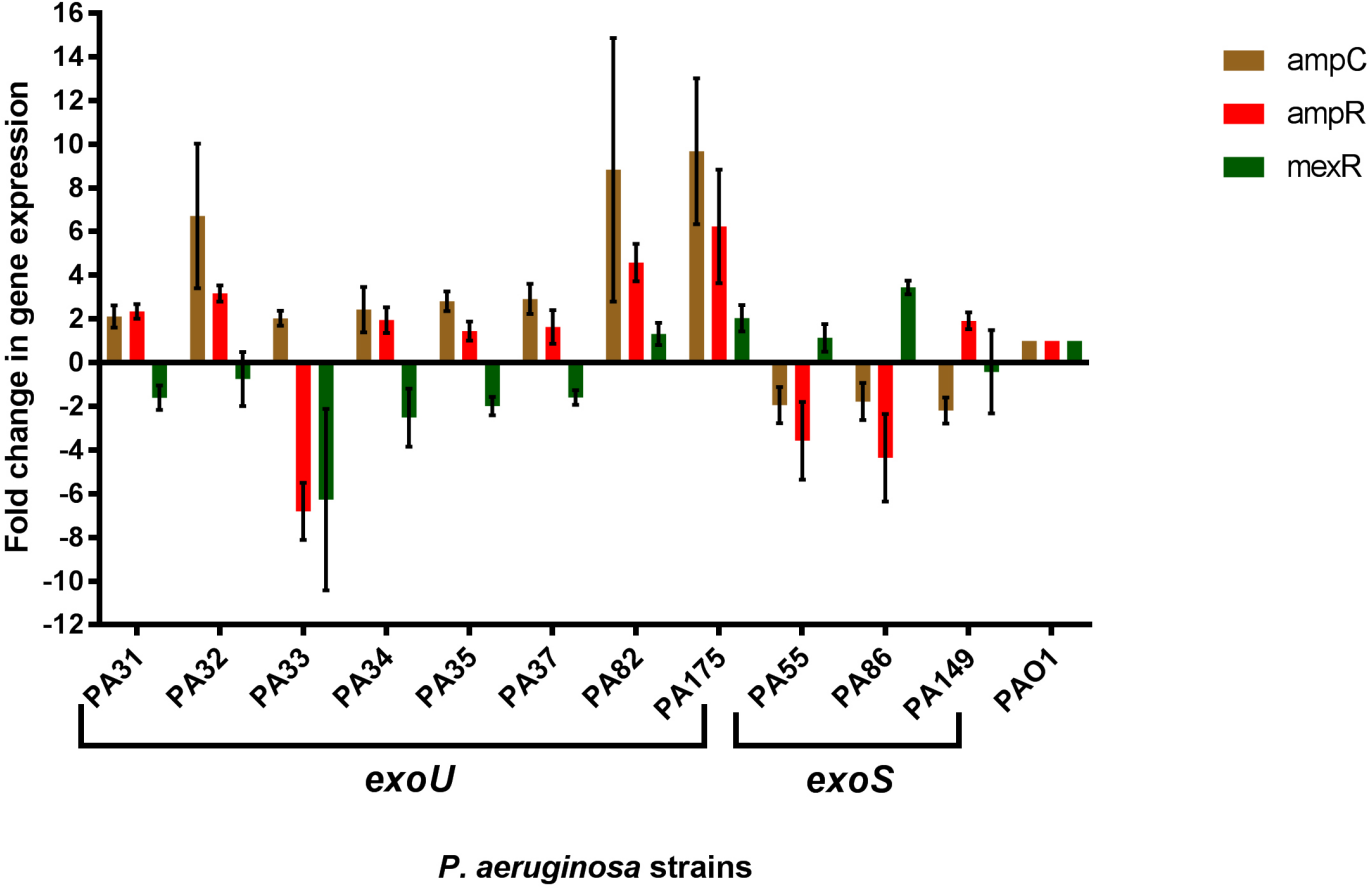
Expression of cephalosporinase (*ampC*) its regulator (*ampR*) and the efflux pump MexAB-OprM regulator (*mexR*) in strains. The relative expression levels were compared to *Pseudomonas aeruginosa* PAO1 (widetype, non-*exoU* strain) and are presented as fold change in gene expression. Error bars represent standard deviation of the mean fold change.

The relative expression of *ampC*, *ampR* and *mexR* genes in all of the *exoU* strains and three randomly selected *exoS* strains (PA55, PA86 and PA149) were compared to *P*. *aeruginosa* PAO1 to analyse the effect of such mutations on expression (Fig 2). The relative expression of the *ampC* was two to nine fold higher in all of the *exoU* strains and was slightly lower in all three *exoS* strains compared to PAO1. Similarly, the relative expression of the *ampR* was at least two fold higher for five out of eight *exoU* strains. For an *exoU* strain (PA33), the *ampR* gene was repressed six fold. The expression of *mexR* gene was repressed in six *exoU* strains while overexpression of *mexR* was observed in two *exoS* strains relative to PAO1.

## Discussion

The *exoU* gene is commonly found in *P*. *aeruginosa* strains isolated from contact lens-related microbial keratitis, at frequencies of 46–54%, [11] whereas it only occurs in 0–14% of non-ocular isolates [6, 22–24]. Similar to a previous report, [10] *exoU*+ strains in the current study had higher resistance to beta-lactams than *exoS*+ strains (100% *exoU* strains vs. 61% *exoS* strains were resistant to at least one beta-lactam). ExoU secreting *P*. *aeruginosa* had more mutations in genes that are associated with beta-lactam resistance (*mexR*, *ampC* and *ampR*) than did *exoS*+ strains. Gene expression analysis suggested that such mutations generally lead to antibiotic resistance, as the expression of *ampC* and *ampR* generally increased while the expression of *mexR* was decreased, compared to the sensitive strain PAO1.

Several *in vivo* and *in vitro* studies have shown that the *exoU* carrying *P*. *aeruginosa* is associated with severe outcome of diseases [7, 12, 20, 21, 43, 44]. In additions, results of this and other studies confirmed that *exoS*+ and *exoU*+ strains have different antibiotic resistance patterns [10, 45]. Therefore, they may require different treatment strategies. Knowing the virulence gene profiles, the clinical outcome of the patients the resistance patterns might be predicted, and this information could be used in deciding appropriate antibiotic treatment.

A mutation at amino acid position 126 (changing valine to glutamic acid) in MexR was common in all *exoU*+ strains. Underexpression of *mexR* has been associated with antibiotic resistance in *P*. *aeruginosa* [46]. Mutations in *mexR* contribute to the over-expression of the MexAB-OprM efflux pump, [47, 48] which in turn is specific to increased resistance to beta-lactams [49]. The current study demonstrated that mutation in *mexR* was correlated with lower transcription of this gene in 75% of *exoU*+ strains. The higher expression of *mexR* in *exoS*+ strains observed here appears to support the hypothesis that possession of the *exoU* gene is associated with beta-lactam resistance.

Various different mutations in *ampC* and *ampR* between *exoU* and *exoS* subpopulations were revealed in the current study. Mutations in *ampC* and *ampR* were more common in *exoU*+ strains. Berrazeg *et al* [37] demonstrated that mutations in *ampC* at amino acid positions G27D, R79Q, T105A, Q156R, L176R, V205L, and G391A were not correlated with beta-lactam resistance and hence were excluded here from analysis in the current study. Mutations at amino acid positions Q155I and V356I in *ampC* were observed in *exoU*+ strains, and all these strains had increased gene expression of *ampC*, suggesting that these mutations may be responsible for this reduced expression. A few *exoS*+ strains and one *exoU*+ strain (PA34) did not have such mutations but were resistant to some beta-lactams. Point mutations in *ampR* (at 114, 182, 283, and 288) can also be responsible for beta-lactam resistance [38] and *exoU*+ strain PA34 carried mutations at E114A and G283E. Mutations at G283E and M288R in *ampR* were exclusive to *exoU*+ strains. These mutations were correlated with over-expression of *ampC* and *ampR* in *exoU*+ strains. The precise mechanism by which acquisition of the *exoU* associated genomic island results in these mutations is not known. For resistance of *exoS*+ strains, it is possible that beta-lactam resistance involves other resistance mechanisms, such as the upregulation of efflux systems MexCD-OprJ, MexEF-OprN, and MexXY-OprM, [50] and hence requires further study for elucidation.

Possession of *exoU* was also associated with higher MICs to fluoroquinolones compared to possession of *exoS*, and this has been shown in previous studies [5, 10, 45, 51–53]. Mutations in the QRDRs of target genes topoisomerase II (*gryA* and *gyrB*) and topoisomerase IV (*parC* and *parE*) have been previously shown to increase fluoroquinolone resistance in *P*. *aeruginosa* [54, 55]. Here, it was also observed that fluoroquinolone resistance in *exoU* strains was correlated with a combination of mutations in *gyrA* and *parC*. Sequence analysis indicated that six out of eight *exoU* strains had at least one mutation in either *gyrA* (T83I) or *parC* (S87L). Consistent with other studies, [54, 56] such mutations were responsible for very high MICs to fluoroquinolones. This suggests the possibility that more virulent strains of *P*. *aeruginosa* that have the *exoU* gene may evolve in the clinical environment where high concentrations of fluoroquinolones are used for treatment; for example, in eye infections [57, 58]. The conditions that favour selection of *exoU*+ strains might result in increased resistance to other antibiotics. In addition, this study also detected several mutations in QRDRs in the *exoS*+ population. Mutations at position E468D of *gyrB* and A473V of *parE* were associated with increase MICs to all tested fluoroquinolones. Such mutations have been previously associated with fluoroquinolone resistance *P*. *aeruginosa* [59, 60]. It appears that different types of mutations in QRDRs evolved in the *exoU*+ and *exoS*+ strains.

An *exoS*+ strain (PA55) was resistant to all beta lactams except aztreonam but no mutations were observed in the studied genes and expression of genes did not correlate with phenotypic resistance. Furthermore, an *exoU*+ strain (PA82) did not have any mutations in QRDRs but was resistant to ciprofloxacin and levofloxacin. However, mutations in V126E the *mexR* of PA82 has been associated with resistance to fluoroquinolone [26, 61]. The evidence from the current study suggests that mutations A110T (PA34) and K76Q (PA175) in *mexR* may confer susceptibility to ceftazidime even in presence of the V126E mutation. This needs to be confirmed by further study.

In addition, we observed a link between the *exoU* and the origin of the strains in India because only one Australian strain (PA175) possessed the *exoU* gene and the resistance rate was higher in the cohort of Indian strains. However, it should be noted that the possession of *exoU* is highly correlated with antibiotic resistance in *P*. *aeruginosa* regardless of source and geographical site of isolation [5, 9, 10, 13, 45]. *ExoU*+ strains may have an evolutionary advantage by having the potential to be both more resistant and more virulent. This is supported by a study that showed higher prevalence of the *exoU* in isolates collected from the hospital environment [62]. A correlation between the geographic origin and the *exoU* carriage was observed in this study potentially due to the relatively unregulated use of antibiotics in India compared to Australia [63]. However, these observations require confirmation with a larger sample size that should include isolates from various sources and a study of associated epidemiological data.

In conclusion, *exoU* carrying strains, which are common in ocular isolates, showed different antibiotic resistance pattern from isolates with *exoS* genotype. The *exoS*+ strains may be protected from the action of antibiotics due to their ability to cause mammalian cells to ingest them (so-called invasive strains). Their residence inside mammalian cells may offer protection from antibiotics and so diminish selection pressure to convert to antibiotic resistance. The *exoU*+ strains had more mutations in drug resistance determining genes (*gyrA*, *parC*, *mexR*, *ampC* and *ampR*), which was likely to be the cause of higher antibiotic resistance in *exoU*+ strains. Differences in mutational rate in two different virulent genotypes indicate more virulent strains can favourably be evolved in the antibiotic rich environment. Therefore, understanding of both virulence traits and antibiotic resistance is essential for more effective prevention of antibiotic resistance.

## Supporting information

S1 Table Variants of *ampC* gene. (PDF)

## References

[1] Richards MJ, Edwards JR, Culver DH, Gaynes RP. Nosocomial infections in medical intensive care units in the United States. National Nosocomial Infections Surveillance System. Critical care medicine. 1999;27(5):887–92. . 10362409 10.1097/00003246-199905000-00020

[2] Yahr TL, Parsek MR. Pseudomonas aeruginosa. Prokaryotes: A Handbook on the Biology of Bacteria, Vol 6, Third Edition. 2006:704–13. 10.1007/0-387-30746-x_22 WOS:000268343700022.

[3] Ding C, Yang Z, Wang J, Liu X, Cao Y, Pan Y, et al Prevalence of *Pseudomonas aeruginosa* and antimicrobial-resistant *Pseudomonas aeruginosa* in patients with pneumonia in mainland China: a systematic review and meta-analysis. International Journal of Infectious Diseases. 2016;49:119–28. 10.1016/j.ijid.2016.06.014. 27329135

[4] Heidary Z, Bandani E, Eftekhary M, Jafari AA. Virulence Genes Profile of Multidrug Resistant *Pseudomonas aeruginosa* Isolated from Iranian Children with UTIs. Acta medica Iranica. 2016;54(3):201–10. Epub 2016/04/25. . 27107526

[5] Cho HH, Kwon KC, Kim S, Koo SH. Correlation between virulence genotype and fluoroquinolone resistance in carbapenem-resistant *Pseudomonas aeruginosa*. Annals of laboratory medicine. 2014;34(4):286–92. Epub 2014/07/02. 10.3343/alm.2014.34.4.286 ; PubMed Central PMCID: PMCPMC4071185. 24982833 PMC4071185

[6] Georgescu M, Gheorghe I, Curutiu C, Lazar V, Bleotu C, Chifiriuc MC. Virulence and resistance features of *Pseudomonas aeruginosa* strains isolated from chronic leg ulcers. BMC infectious diseases. 2016;16 Suppl 1:92 Epub 2016/05/14. 10.1186/s12879-016-1396-3 ; PubMed Central PMCID: PMCPMC4890939. 27169367 PMC4890939

[7] Sawa T, Shimizu M, Moriyama K, Wiener-Kronish JP. Association between *Pseudomonas aeruginosa* type III secretion, antibiotic resistance, and clinical outcome: a review. Critical care (London, England). 2014;18(6):668 Epub 2015/02/13. 10.1186/s13054-014-0668-9 ; PubMed Central PMCID: PMCPMC4331484. 25672496 PMC4331484

[8] Tran QT, Nawaz MS, Deck J, Foley S, Nguyen K, Cerniglia CE. Detection of type III secretion system virulence and mutations in gyrA and parC genes among quinolone-resistant strains of *Pseudomonas aeruginosa* isolated from imported shrimp. Foodborne pathogens and disease. 2011;8(3):451–3. Epub 2010/12/02. 10.1089/fpd.2010.0687 . 21117986

[9] Agnello M, Finkel SE, Wong-Beringer A. Fitness Cost of Fluoroquinolone Resistance in Clinical Isolates of *Pseudomonas aeruginosa* Differs by Type III Secretion Genotype. Frontiers in microbiology. 2016;7:1591 Epub 2016/10/21. 10.3389/fmicb.2016.01591 ; PubMed Central PMCID: PMCPMC5047889. 27757111 PMC5047889

[10] Garey KW, Vo QP, Larocco MT, Gentry LO, Tam VH. Prevalence of type III secretion protein exoenzymes and antimicrobial susceptibility patterns from bloodstream isolates of patients with *Pseudomonas aeruginosa* bacteremia. Journal of chemotherapy. 2008;20(6):714–20. 10.1179/joc.2008.20.6.714 . 19129069

[11] Choy MH, Stapleton F, Willcox MD, Zhu H. Comparison of virulence factors in *Pseudomonas aeruginosa* strains isolated from contact lens- and non-contact lens-related keratitis. Journal of medical microbiology. 2008;57(Pt 12):1539–46. 10.1099/jmm.0.2008/003723-0 . 19018027

[12] Finck-Barbancon V, Goranson J, Zhu L, Sawa T, Wiener-Kronish JP, Fleiszig SM, et al ExoU expression by *Pseudomonas aeruginosa* correlates with acute cytotoxicity and epithelial injury. Molecular microbiology. 1997;25(3):547–57. Epub 1997/08/01. . 9302017 10.1046/j.1365-2958.1997.4891851.x

[13] Agnello M, Wong-Beringer A. Differentiation in Quinolone Resistance by Virulence Genotype in *Pseudomonas aeruginosa*. PloS one. 2012;7(8). 10.1371/journal.pone.0042973 ; PubMed Central PMCID: PMCPMC3414457. 22905192 PMC3414457

[14] Sato H, Frank DW, Hillard CJ, Feix JB, Pankhaniya RR, Moriyama K, et al The mechanism of action of the *Pseudomonas aeruginosa*-encoded type III cytotoxin, ExoU. The EMBO journal. 2003;22(12):2959–69. 10.1093/emboj/cdg290 ; PubMed Central PMCID: PMCPMC162142. 12805211 PMC162142

[15] Hauser AR. The type III secretion system of *Pseudomonas aeruginosa*: infection by injection. Nature reviews Microbiology. 2009;7(9):654–65. 10.1038/nrmicro2199 ; PubMed Central PMCID: PMCPMC2766515. 19680249 PMC2766515

[16] Berthelot P, Attree I, Plesiat P, Chabert J, de Bentzmann S, Pozzetto B, et al Genotypic and phenotypic analysis of type III secretion system in a cohort of *Pseudomonas aeruginosa* bacteremia isolates: evidence for a possible association between O serotypes and exo genes. The Journal of infectious diseases. 2003;188(4):512–8. Epub 2003/08/05. 10.1086/377000 . 12898437

[17] Fleiszig SM, Wiener-Kronish JP, Miyazaki H, Vallas V, Mostov KE, Kanada D, et al *Pseudomonas aeruginosa*-mediated cytotoxicity and invasion correlate with distinct genotypes at the loci encoding exoenzyme S. Infection and immunity. 1997;65(2):579–86. ; PubMed Central PMCID: PMCPMC176099. 9009316 10.1128/iai.65.2.579-586.1997 PMC176099

[18] Kulasekara BR, Kulasekara HD, Wolfgang MC, Stevens L, Frank DW, Lory S. Acquisition and evolution of the exoU locus in *Pseudomonas aeruginosa*. Journal of bacteriology. 2006;188(11):4037–50. 10.1128/JB.02000-05 ; PubMed Central PMCID: PMCPMC1482899. 16707695 PMC1482899

[19] Wolfgang MC, Kulasekara BR, Liang X, Boyd D, Wu K, Yang Q, et al Conservation of genome content and virulence determinants among clinical and environmental isolates of *Pseudomonas aeruginosa*. Proceedings of the National Academy of Sciences of the United States of America. 2003;100(14):8484–9. 10.1073/pnas.0832438100 ; PubMed Central PMCID: PMCPMC166255. 12815109 PMC166255

[20] Hauser AR, Cobb E, Bodi M, Mariscal D, Valles J, Engel JN, et al Type III protein secretion is associated with poor clinical outcomes in patients with ventilator-associated pneumonia caused by *Pseudomonas aeruginosa*. Critical care medicine. 2002;30(3):521–8. . 11990909 10.1097/00003246-200203000-00005

[21] Stewart RM, Wiehlmann L, Ashelford KE, Preston SJ, Frimmersdorf E, Campbell BJ, et al Genetic characterization indicates that a specific subpopulation of *Pseudomonas aeruginosa* is associated with keratitis infections. Journal of clinical microbiology. 2011;49(3):993–1003. 10.1128/JCM.02036-10 ; PubMed Central PMCID: PMCPMC3067716. 21227987 PMC3067716

[22] Yamaguchi S, Suzuki T, Kobayashi T, Oka N, Ishikawa E, Shinomiya H, et al Genotypic analysis of *Pseudomonas aeruginosa* isolated from ocular infection. Journal of infection and chemotherapy : official journal of the Japan Society of Chemotherapy. 2014;20(7):407–11. Epub 2014/04/22. 10.1016/j.jiac.2014.02.007 . 24746897

[23] Tingpej P, Smith L, Rose B, Zhu H, Conibear T, Al Nassafi K, et al Phenotypic characterization of clonal and nonclonal *Pseudomonas aeruginosa* strains isolated from lungs of adults with cystic fibrosis. Journal of clinical microbiology. 2007;45(6):1697–704. 10.1128/JCM.02364-06 ; PubMed Central PMCID: PMCPMC1933084. 17392437 PMC1933084

[24] de Almeida Silva KCF, Calomino MA, Deutsch G, de Castilho SR, de Paula GR, Esper LMR, et al Molecular characterization of multidrug-resistant (MDR) *Pseudomonas aeruginosa* isolated in a burn center. Burns : journal of the International Society for Burn Injuries. 2017;43(1):137–43. Epub 2016/09/07. 10.1016/j.burns.2016.07.002 . 27595453

[25] Jacoby GA. AmpC beta-lactamases. Clinical microbiology reviews. 2009;22(1):161–82, Table of Contents. 10.1128/CMR.00036-08 ; PubMed Central PMCID: PMC2620637. 19136439 PMC2620637

[26] Poole K. Multidrug efflux pumps and antimicrobial resistance in *Pseudomonas aeruginosa* and related organisms. Journal of molecular microbiology and biotechnology. 2001;3(2):255–64. . 11321581

[27] Clinical and Laboratory Standards Institute(CLSI). Methods for Dilution Antimicrobial Susceptibility Tests for Bacteria That Grow Aerobically; Approved Standard—Ninth Edition CLSI 2012;32(M07-A9). Wayne, PA.

[28] Wiegand I, Hilpert K, Hancock RE. Agar and broth dilution methods to determine the minimal inhibitory concentration (MIC) of antimicrobial substances. Nature protocols. 2008;3(2):163–75. 10.1038/nprot.2007.521 . 18274517

[29] Clinical and Laboratory Standards Institute(CLSI). Performance standards for antimicrobial susceptibility testing; twenty-second information supplement. CLSI document M100-S22. 2012;32(3). Wayne, PA.

[30] European Committee on Antimicrobial Susceptibility Testing (EUCAST). Breakpoint tables for interpretation of MICs and zone diameters. Version 6.0: The European Committee on Antimicrobial Susceptibility Testing; 2016. Available from: http://www.eucast.org/clinical_breakpoints/.

[31] Bankevich A, Nurk S, Antipov D, Gurevich AA, Dvorkin M, Kulikov AS, et al SPAdes: a new genome assembly algorithm and its applications to single-cell sequencing. J Comput Biol. 2012;19(5):455–77. 10.1089/cmb.2012.0021 ; PubMed Central PMCID: PMCPMC3342519. 22506599 PMC3342519

[32] Seemann T. Prokka: rapid prokaryotic genome annotation. Bioinformatics (Oxford, England). 2014;30(14):2068–9. 10.1093/bioinformatics/btu153 . 24642063

[33] Langmead B, Salzberg SL. Fast gapped-read alignment with Bowtie 2. Nat Methods. 2012;9(4):357–9. 10.1038/nmeth.1923 ; PubMed Central PMCID: PMCPMC3322381. 22388286 PMC3322381

[34] Li H, Handsaker B, Wysoker A, Fennell T, Ruan J, Homer N, et al The Sequence Alignment/Map format and SAMtools. Bioinformatics (Oxford, England). 2009;25(16):2078–9. 10.1093/bioinformatics/btp352 ; PubMed Central PMCID: PMCPMC2723002. 19505943 PMC2723002

[35] Cingolani P, Platts A, Wang le L, Coon M, Nguyen T, Wang L, et al A program for annotating and predicting the effects of single nucleotide polymorphisms, SnpEff: SNPs in the genome of Drosophila melanogaster strain w1118; iso-2; iso-3. Fly. 2012;6(2):80–92. Epub 2012/06/26. 10.4161/fly.19695 ; PubMed Central PMCID: PMCPMC3679285. 22728672 PMC3679285

[36] Bruchmann S, Dotsch A, Nouri B, Chaberny IF, Haussler S. Quantitative contributions of target alteration and decreased drug accumulation to *Pseudomonas aeruginosa* fluoroquinolone resistance. Antimicrobial agents and chemotherapy. 2013;57(3):1361–8. 10.1128/AAC.01581-12 ; PubMed Central PMCID: PMCPMC3591863. 23274661 PMC3591863

[37] Berrazeg M, Jeannot K, Ntsogo Enguéné VY, Broutin I, Loeffert S, Fournier D, et al Mutations in β-Lactamase AmpC Increase Resistance of *Pseudomonas aeruginosa* Isolates to Antipseudomonal Cephalosporins. Antimicrobial agents and chemotherapy. 2015;59(10):6248–55. 10.1128/AAC.00825-15 ; PubMed Central PMCID: PMCPMC4576058. 26248364 PMC4576058

[38] Tam VH, Schilling AN, LaRocco MT, Gentry LO, Lolans K, Quinn JP, et al Prevalence of AmpC over-expression in bloodstream isolates of *Pseudomonas aeruginosa*. Clinical microbiology and infection : the official publication of the European Society of Clinical Microbiology and Infectious Diseases. 2007;13(4):413–8. 10.1111/j.1469-0691.2006.01674.x . 17359326

[39] Vaez H, Faghri J, Isfahani BN, Moghim S, Yadegari S, Fazeli H, et al Efflux pump regulatory genes mutations in multidrug resistance *Pseudomonas aeruginosa* isolated from wound infections in Isfahan hospitals. Adv Biomed Res. 2014;3:117 10.4103/2277-9175.133183 ; PubMed Central PMCID: PMCPMC4063115. 24949288 PMC4063115

[40] Hocquet D, Bertrand X, Kohler T, Talon D, Plesiat P. Genetic and phenotypic variations of a resistant *Pseudomonas aeruginosa* epidemic clone. Antimicrobial agents and chemotherapy. 2003;47(6):1887–94. 10.1128/AAC.47.6.1887-1894.2003 ; PubMed Central PMCID: PMC155826. 12760863 PMC155826

[41] Walker SE, Lorsch J. Chapter Nineteen—RNA Purification–Precipitation Methods In: Lorsch J, editor. Methods in enzymology. 530: Academic Press; 2013 p. 337–43. 10.1016/B978-0-12-420037-1.00019-1 24034331

[42] Schmittgen TD, Livak KJ. Analyzing real-time PCR data by the comparative CT method. Nature protocols. 2008;3(6):1101–8. 10.1038/nprot.2008.73 18546601

[43] Hsu DI, Okamoto MP, Murthy R, Wong-Beringer A. Fluoroquinolone-resistant *Pseudomonas aeruginosa*: risk factors for acquisition and impact on outcomes. The Journal of antimicrobial chemotherapy. 2005;55(4):535–41. 10.1093/jac/dki026 . 15728150

[44] Fleiszig SM, Lee EJ, Wu C, Andika RC, Vallas V, Portoles M, et al Cytotoxic strains of *Pseudomonas aeruginosa* can damage the intact corneal surface in vitro. The CLAO journal : official publication of the Contact Lens Association of Ophthalmologists, Inc. 1998;24(1):41–7. . 9474453

[45] Wong-Beringer A, Wiener-Kronish J, Lynch S, Flanagan J. Comparison of type III secretion system virulence among fluoroquinolone-susceptible and -resistant clinical isolates of *Pseudomonas aeruginosa*. Clinical microbiology and infection : the official publication of the European Society of Clinical Microbiology and Infectious Diseases. 2008;14(4):330–6. 10.1111/j.1469-0691.2007.01939.x . 18190571

[46] Dumas JL, van Delden C, Perron K, Kohler T. Analysis of antibiotic resistance gene expression in *Pseudomonas aeruginosa* by quantitative real-time-PCR. FEMS microbiology letters. 2006;254(2):217–25. Epub 2006/02/01. 10.1111/j.1574-6968.2005.00008.x . 16445748

[47] Saito K, Yoneyama H, Nakae T. nalB-type mutations causing the overexpression of the MexAB-OprM efflux pump are located in the mexR gene of the *Pseudomonas aeruginosa* chromosome. FEMS microbiology letters. 1999;179(1):67–72. . 10481088 10.1111/j.1574-6968.1999.tb08709.x

[48] Adewoye L, Sutherland A, Srikumar R, Poole K. The mexR repressor of the mexAB-oprM multidrug efflux operon in *Pseudomonas aeruginosa*: characterization of mutations compromising activity. Journal of bacteriology. 2002;184(15):4308–12. 10.1128/JB.184.15.4308-4312.2002 ; PubMed Central PMCID: PMCPMC135222. 12107151 PMC135222

[49] Masuda N, Sakagawa E, Ohya S, Gotoh N, Tsujimoto H, Nishino T. Substrate specificities of MexAB-OprM, MexCD-OprJ, and MexXY-oprM efflux pumps in *Pseudomonas aeruginosa*. Antimicrobial agents and chemotherapy. 2000;44(12):3322–7. ; PubMed Central PMCID: PMCPMC90200. 11083635 10.1128/aac.44.12.3322-3327.2000 PMC90200

[50] Poole K. Efflux-mediated antimicrobial resistance. The Journal of antimicrobial chemotherapy. 2005;56(1):20–51. 10.1093/jac/dki171 . 15914491

[51] Park MH, Kim SY, Roh EY, Lee HS. Difference of Type 3 secretion system (T3SS) effector gene genotypes (exoU and exoS) and its implication to antibiotics resistances in isolates of *Pseudomonas aeruginosa* from chronic otitis media. Auris, nasus, larynx. 2017;44(3):258–65. Epub 2016/07/28. 10.1016/j.anl.2016.07.005 . 27461174

[52] Borkar DS, Acharya NR, Leong C, Lalitha P, Srinivasan M, Oldenburg CE, et al Cytotoxic clinical isolates of *Pseudomonas aeruginosa* identified during the Steroids for Corneal Ulcers Trial show elevated resistance to fluoroquinolones. BMC ophthalmology. 2014;14:54 Epub 2014/04/26. 10.1186/1471-2415-14-54 ; PubMed Central PMCID: PMCPMC4008435. 24761794 PMC4008435

[53] Mitov I, Strateva T, Markova B. Prevalence of Virulence Genes Among Bulgarian Nosocomial and Cystic Fibrosis Isolates of *Pseudomonas aeruginosa*. Brazilian Journal of Microbiology. 2010;41(3):588–95. 10.1590/S1517-83822010000300008 ; PubMed Central PMCID: PMCPMC3768660. 24031533 PMC3768660

[54] Ling JM, Chan EW, Lam AW, Cheng AF. Mutations in Topoisomerase Genes of Fluoroquinolone-Resistant Salmonellae in Hong Kong. Antimicrobial agents and chemotherapy. 2003;47(11):3567–73. 10.1128/AAC.47.11.3567-3573.2003 ; PubMed Central PMCID: PMCPMC253778. 14576119 PMC253778

[55] Lee JK, Lee YS, Park YK, Kim BS. Alterations in the GyrA and GyrB subunits of topoisomerase II and the ParC and ParE subunits of topoisomerase IV in ciprofloxacin-resistant clinical isolates of *Pseudomonas aeruginosa*. International journal of antimicrobial agents. 2005;25(4):290–5. Epub 2005/03/24. 10.1016/j.ijantimicag.2004.11.012 . 15784307

[56] Hirose K, Hashimoto A, Tamura K, Kawamura Y, Ezaki T, Sagara H, et al DNA sequence analysis of DNA gyrase and DNA topoisomerase IV quinolone resistance-determining regions of Salmonella enterica serovar Typhi and serovar Paratyphi A. Antimicrobial agents and chemotherapy. 2002;46(10):3249–52. 10.1128/AAC.46.10.3249-3252.2002 ; PubMed Central PMCID: PMC128770. 12234852 PMC128770

[57] Green M, Apel A, Stapleton F. Risk factors and causative organisms in microbial keratitis. Cornea. 2008;27(1):22–7. 10.1097/ICO.0b013e318156caf2 . 18245962

[58] Keay L, Edwards K, Stapleton F. Referral pathways and management of contact lens-related microbial keratitis in Australia and New Zealand. Clinical & experimental ophthalmology. 2008;36(3):209–16. 10.1111/j.1442-9071.2008.01722.x . 18412588

[59] Lee JK, Lee YS, Park YK, Kim BS. Alterations in the GyrA and GyrB subunits of topoisomerase II and the ParC and ParE subunits of topoisomerase IV in ciprofloxacin-resistant clinical isolates of *Pseudomonas aeruginosa*. International journal of antimicrobial agents. 2005;25(4):290–5. 10.1016/j.ijantimicag.2004.11.012. 15784307

[60] Akasaka T, Tanaka M, Yamaguchi A, Sato K. Type II topoisomerase mutations in fluoroquinolone-resistant clinical strains of *Pseudomonas aeruginosa* isolated in 1998 and 1999: role of target enzyme in mechanism of fluoroquinolone resistance. Antimicrobial agents and chemotherapy. 2001;45(8):2263–8. 10.1128/AAC.45.8.2263-2268.2001 ; PubMed Central PMCID: PMC90640. 11451683 PMC90640

[61] Choudhury D, Ghosh A, Dhar Chanda D, Das Talukdar A, Dutta Choudhury M, Paul D, et al Premature Termination of MexR Leads to Overexpression of MexAB-OprM Efflux Pump in *Pseudomonas aeruginosa* in a Tertiary Referral Hospital in India. PloS one. 2016;11(2):e0149156 10.1371/journal.pone.0149156 26866484 PMC4750933

[62] Bradbury RS, Roddam LF, Merritt A, Reid DW, Champion AC. Virulence gene distribution in clinical, nosocomial and environmental isolates of *Pseudomonas aeruginosa*. Journal of medical microbiology. 2010;59(Pt 8):881–90. Epub 2010/05/01. 10.1099/jmm.0.018283-0 . 20430902

[63] Laxminarayan R, Matsoso P, Pant S, Brower C, Røttingen J-A, Klugman K, et al Access to effective antimicrobials: a worldwide challenge. The Lancet. 2016;387(10014):168–75. 10.1016/S0140-6736(15)00474-2. 26603918

[64] Stover CK, Pham XQ, Erwin AL, Mizoguchi SD, Warrener P, Hickey MJ, et al Complete genome sequence of *Pseudomonas aeruginosa* PAO1, an opportunistic pathogen. Nature. 2000;406(6799):959–64. 10.1038/35023079 . 10984043

