# Supplementary material for: Association between possession of ExoU and antibiotic resistance in Pseudomonas aeruginosa
Source: PLoS One. 2018 Sep 28;13(9):e0204936. doi: 10.1371/journal.pone.0204936 (PMC6161911; doi:10.1371/journal.pone.0204936)
Supplement: S1 Table — (PDF) [file pone.0204936.s001.pdf]

Supplementary Table S1: Variants of *ampC* gene

Strains	ampC variants
**PA17**	Thr105Ala
**PA31**	Gly27Asp<br>Thr105Ala<br>Val205Leu<br>Val356Ile<br>Gly391Ala
**PA32**	Gly27Asp<br>Thr105Ala<br>Val205Leu<br>Val356Ile<br>Gly391Ala
**PA33**	Gly27Asp<br>Gly391Ala<br>Thr105Ala<br>Val205Leu<br>Val356Ile
**PA34**	Thr105Ala<br>Val205Leu<br>Gly391Ala
**PA35**	Gly27Asp<br>Thr105Ala<br>Val205Leu<br>Val356Ile<br>Gly391Ala
**PA37**	Gly27Asp<br>Thr105Ala<br>Val205Leu<br>Val356Ile<br>Gly391Ala
**PA40**	Arg79Gln<br>Thr105Ala
**PA149**	Arg79Gln<br>Thr105Ala
**PA157**	Thr105Ala<br>Leu176Arg
**PA171**	Arg79Gln<br>Thr105Ala
**PA175**	Gly27Asp<br>Thr105Ala<br>Val205Leu<br>Val356Ile<br>Gly391Ala
**PA55**	No variants
**PA57**	Arg5Gly<br>Thr21Ala<br>Pro23Ser<br>Ala36Thr

	Thr105Ala<br>Gln117Leu<br>Ala170Thr<br>Leu200Ile<br>Val205Leu<br>Arg273Lys<br>Val356Ile<br>Gly391Ala
**PA59**	Thr105Ala
**PA64**	Thr105Ala
**PA66**	Thr105Ala
**PA82**	Pro7Ser<br>Gly391Ala<br>Val205Leu<br>Gln155Arg<br>Thr105Ala<br>Gly27Asp
**PA86**	Thr105Ala
**PA92**	Thr105Ala
**PA100**	Arg79Gln<br>Thr105Ala
**PA102**	Arg79Gln<br>Thr105Ala
